# Supplementary material for: Isolation and Identification of Alternaria alternata from Potato Plants Affected by Leaf Spot Disease in Korea: Selection of Effective Fungicides
Source: J Fungi (Basel). 2024 Jan 7;10(1):53. doi: 10.3390/jof10010053 (PMC10820076; doi:10.3390/jof10010053)
Supplement: Supplementary file 1 [file jof-10-00053-s001.zip › Supplementary Figure S6.pdf]

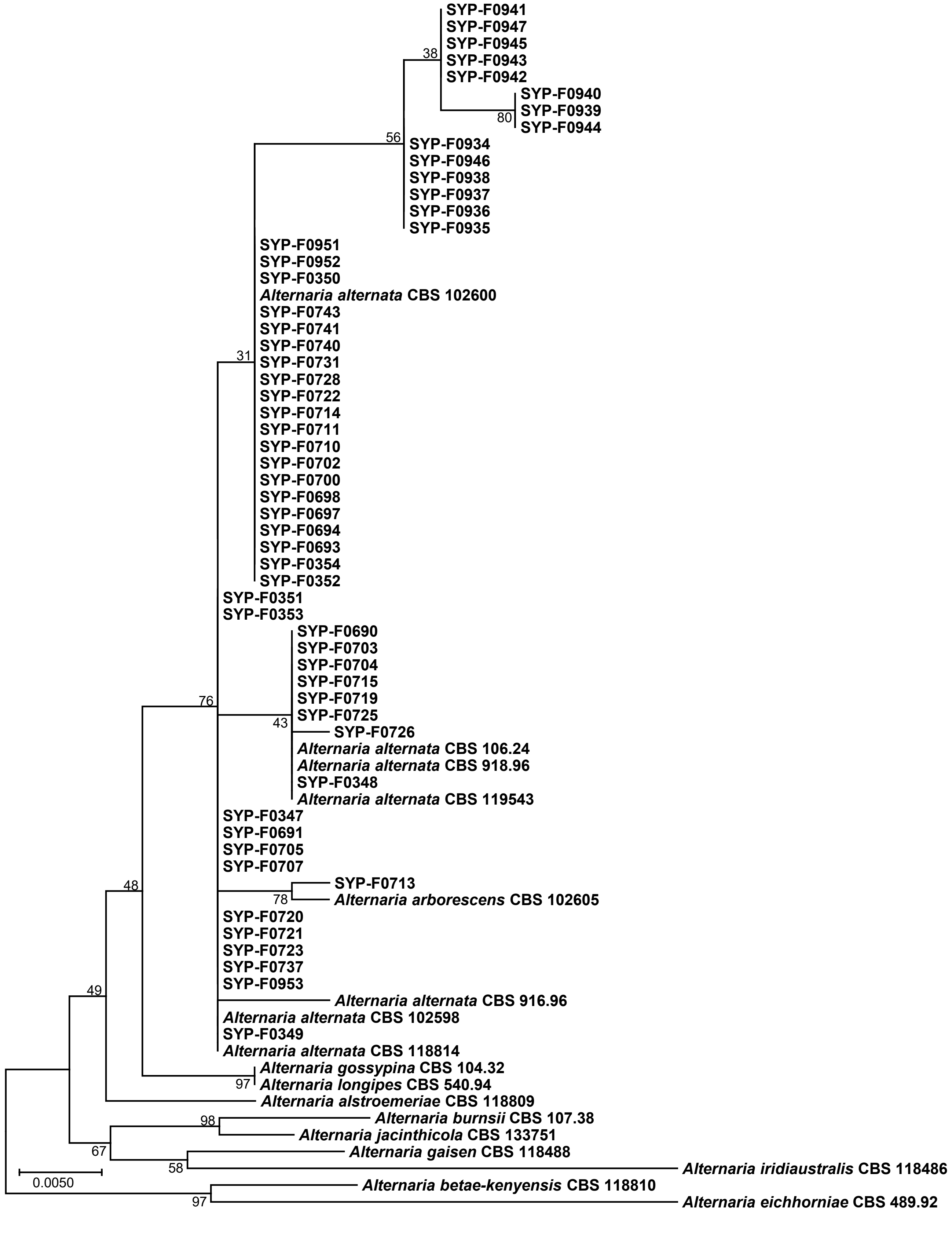

Supplementary Figure S6. Phylogenetic tree constructed based on the *endoPG* gene of the *Alternaria* spp. isolates and reference sequences retrieved from GenBank (accession numbers shown in Supplementary Table S1). The tree was constructed by maximum-likelihood, and bootstrap values (1,000 replications) are shown in front of each node. MEGA X was used for the analysis.
