# Supplementary material for: Isolation and Identification of Alternaria alternata from Potato Plants Affected by Leaf Spot Disease in Korea: Selection of Effective Fungicides
Source: J Fungi (Basel). 2024 Jan 7;10(1):53. doi: 10.3390/jof10010053 (PMC10820076; doi:10.3390/jof10010053)
Supplement: Supplementary file 1 [file jof-10-00053-s001.zip › Supplementary Table S1_NCBI blast result_Accession nos.pdf]

Supplementary Table S1. Isolates used in this study and their GenBank accession numbers. Bold accession numbers were generated in other studies

Isolates	The closest matched GenBank taxa	Acession numbers of gene bank						
		ITS	GAPDH	TEF1	RPB2	Alta1	endoPG	OPA10-2
SYP-F0347	*Alternaria alternata*	OR885731	OR900248	OR910019	OR916211	OR900374	OR901696	OR900460
SYP-F0348	*Alternaria alternata*	OR885732	OR900249	OR910020	OR916212	OR900375	OR901697	OR900461
SYP-F0349	*Alternaria alternata*	OR885733	OR900250	OR910021	OR916213	OR900376	OR901698	OR900462
SYP-F0350	*Alternaria alternata*	OR885734	OR900251	OR910022	OR916214	OR900377	OR901699	OR900463
SYP-F0351	*Alternaria alternata*	OR885735	OR900252	OR910023	OR916215	OR900378	OR901700	OR900464
SYP-F0354	*Alternaria alternata*	OR885738	OR900253	OR910024	OR916216	OR900379	OR901701	OR900465
SYP-F0690	*Alternaria alternata*	OR885739	OR900254	OR910025	OR916217	OR900380	OR901702	OR900466
SYP-F0691	*Alternaria alternata*	OR885740	OR900255	OR910026	OR916218	OR900381	OR901703	OR900467
SYP-F0693	*Alternaria alternata*	OR885741	OR900256	OR910027	OR916219	OR900382	OR901704	OR900468
SYP-F0694	*Alternaria alternata*	OR885742	OR900257	OR910028	OR916220	OR900383	OR901705	OR900469
SYP-F0697	*Alternaria alternata*	OR885743	OR900258	OR910029	OR916221	OR900384	OR901706	OR900470
SYP-F0698	*Alternaria alternata*	OR885744	OR900259	OR910030	OR916222	OR900385	OR901707	OR900471
SYP-F0700	*Alternaria alternata*	OR885745	OR900260	OR910031	OR916223	OR900386	OR901708	OR900472
SYP-F0702	*Alternaria alternata*	OR885747	OR900261	OR910032	OR916224	OR900387	OR901709	OR900473
SYP-F0703	*Alternaria alternata*	OR885748	OR900262	OR910033	OR916225	OR900388	OR901710	OR900474
SYP-F0704	*Alternaria alternata*	OR885749	OR900263	OR910034	OR916226	OR900389	OR901711	OR900475
SYP-F0705	*Alternaria alternata*	OR885750	OR900264	OR910035	OR916227	OR900390	OR901712	OR900476
SYP-F0707	*Alternaria alternata*	OR885752	OR900265	OR910036	OR916228	OR900391	OR901713	OR900477
SYP-F0710	*Alternaria alternata*	OR885753	OR900266	OR910037	OR916229	OR900392	OR901714	OR900478
SYP-F0711	*Alternaria alternata*	OR885754	OR900267	OR910038	OR916230	OR900393	OR901715	OR900479
SYP-F0715	*Alternaria alternata*	OR885758	OR900268	OR910039	OR916231	OR900394	OR901716	OR900480
SYP-F0719	*Alternaria alternata*	OR885762	OR900269	OR910040	OR916232	OR900395	OR901717	OR900481
SYP-F0720	*Alternaria alternata*	OR885763	OR900270	OR910041	OR916233	OR900396	OR901718	OR900482
SYP-F0721	*Alternaria alternata*	OR885764	OR900271	OR910042	OR916234	OR900397	OR901719	OR900483
SYP-F0722	*Alternaria alternata*	OR885765	OR900272	OR910043	OR916235	OR900398	OR901720	OR900484
SYP-F0723	*Alternaria alternata*	OR885766	OR900273	OR910044	OR916236	OR900399	OR901721	OR900485

SYP-F0725	*Alternaria alternata*	OR885767	OR900274	OR910045	OR916237	OR900400	OR901722	OR900486
SYP-F0726	*Alternaria alternata*	OR885768	OR900275	OR910046	OR916238	OR900401	OR901723	OR900487
SYP-F0728	*Alternaria alternata*	OR885769	OR900276	OR910047	OR916239	OR900402	OR901724	OR900488
SYP-F0731	*Alternaria alternata*	OR885771	OR900277	OR910048	OR916240	OR900403	OR901725	OR900489
SYP-F0737	*Alternaria alternata*	OR885773	OR900278	OR910049	OR916241	OR900404	OR901726	OR900490
SYP-F0740	*Alternaria alternata*	OR885774	OR900279	OR910050	OR916242	OR900405	OR901727	OR900491
SYP-F0741	*Alternaria alternata*	OR885775	OR900280	OR910051	OR916243	OR900406	OR901728	OR900492
SYP-F0743	*Alternaria alternata*	OR885777	OR900281	OR910052	OR916244	OR912470	OR901729	OR900493
SYP-F0934	*Alternaria alternata*	OP581413	OP588286	OP588300	OP588314	OP588328	OP588342	OP588356
SYP-F0935	*Alternaria alternata*	OP581414	OP588287	OP588301	OP588315	OP588329	OP588343	OP588357
SYP-F0936	*Alternaria alternata*	OP581415	OP588288	OP588302	OP588316	OP588330	OP588344	OP588358
SYP-F0937	*Alternaria alternata*	OR885782	OR900282	OR910053	OR916245	OR900407	OR901730	OR900494
SYP-F0938	*Alternaria alternata*	OR885783	OR900283	OR910054	OR916246	OR900408	OR901731	OR900495
SYP-F0939	*Alternaria alternata*	OR885784	OR900284	OR910055	OR916247	OR900409	OR901732	OR900496
SYP-F0940	*Alternaria alternata*	OR885785	OR900285	OR910056	OR916248	OR900410	OR901733	OR900497
SYP-F0941	*Alternaria alternata*	OR885786	OR900286	OR910057	OR916249	OR900411	OR901734	OR900498
SYP-F0942	*Alternaria alternata*	OR885787	OR900287	OR910058	OR916250	OR900412	OR901735	OR900499
SYP-F0943	*Alternaria alternata*	OR885788	OR900288	OR910059	OR916251	OR900413	OR901736	OR900500
SYP-F0944	*Alternaria alternata*	OR885789	OR900289	OR910060	OR916252	OR900414	OR901737	OR900501
SYP-F0945	*Alternaria alternata*	OR885790	OR900290	OR910061	OR916253	OR900415	OR901738	OR900502
SYP-F0946	*Alternaria alternata*	OR885791	OR900291	OR910062	OR916254	OR900416	OR901739	OR900503
SYP-F0947	*Alternaria alternata*	OR885792	OR900292	OR910063	OR916255	OR900417	OR901740	OR900504
SYP-F0951	*Alternaria alternata*	OR885793	OR900293	OR910064	OR916256	OR900418	OR901741	OR900505
SYP-F0952	*Alternaria alternata*	OR885794	OR900294	OR910065	OR916257	OR900419	OR901742	OR900506
SYP-F0953	*Alternaria alternata*	OR885795	OR900295	OR910066	OR916258	OR900420	OR901743	OR900507
SYP-F0352	*Alternaria alternata*	OR885736	OR900296	OR901687	OR909916	OR900421	OR901744	OR900508
SYP-F0353	*Alternaria alternata*	OR885737	OR900297	OR901688	OR909917	OR900422	OR901745	OR900509
SYP-F0713	*Alternaria alternata*	OR885756	OR900298	OR901689	OR909918	OR900423	OR901746	OR900510
SYP-F0714	*Alternaria alternata*	OR885757	OR900299	OR901690	OR909919	OR900424	OR901747	OR900511

Reference Isolates	*Alternaria alstroemeriae* CBS 118809	KP124297	KP124154	KP125072	KP124765	np	KP123994	KP124602
	Alternaria alternantherae CBS 124392	KC584179	KC584096	KC584633	KC584374	KP123846	np	np
	Alternaria alternata CBS 106.24	KP124298	KP124155	KP125073	KP124766	KP123847	AY295020	JQ800620
	Alternaria alternata CBS 916.96	AF347031	AY278808	KC584634	KC584375	AY563301	JQ811978	KP124632
	Alternaria alternata CBS 918.96	AF347032	AY278809	KC584693	KC584435	AY563302	KP124026	KP124633
	Alternaria alternata CBS 102598	KP124329	KP124184	KP125105	KP124797	KP123878	KP124031	KP124638
	Alternaria alternata CBS 102600	KP124331	KP124186	KP125107	KP124799	KP123880	KP124033	KP124640
	Alternaria alternata CBS 118814	KP124357	KP124211	KP125133	KP124825	KP123906	KP124059	KP124669
	Alternaria alternata CBS 119543	KP124363	KP124215	KP125139	KP124831	KP123911	KP124065	KP124674
	Alternaria arborescens SC_CBS 102605	AF347033	AY278810	KC584636	KC584377	AY563303	AY295028	KP124712
	Alternaria betae-kenyensis CBS 118810	KP124419	KP124270	KP125197	KP124888	KP123966	KP124123	KP124733
	Alternaria burnsii CBS 107.38	KP124420	JQ646305	KP125198	KP124889	KP123967	KP124124	KP124734
	Alternaria eichhorniae CBS 489.92	KC146356	KP124276	KP125204	KP124895	KP123973	KP124130	KP124740
	Alternaria gaisen CBS 118488	KP124427	KP124278	KP125206	KP124897	KP123975	KP124132	KP124743
	Alternaria gossypina CBS 104.32	KP124430	JQ646312	KP125209	KP124900	JQ646395	KP124135	KP124746
	Alternaria iridiaustralis CBS 118486	KP124435	KP124284	KP125214	KP124905	KP123981	KP124140	KP124751
	Alternaria jacinthicola CBS 133751; MUCL 53159	KP124438	KP124287	KP125217	KP124908	KP123984	KP124143	KP124754
	Alternaria longipes CBS 540.94	AY278835	AY278811	KC584667	KC584409	AY563304	KP124147	KP124758
	Alternaria brassicicola CBS 118699	JX499031	KC584103	KC584642	KC584383			
	Alternaria infectoria CBS 210.86	DQ323697	AY278793	KC584662	KC584404			
	Alternaria papavericola CBS 116606	FJ357310	FJ357298	KC584705	KC584446			
	Alternaria avenicola CBS 121459	KC584183	KC584100	KC584639	KC584380			
	Alternaria solani CBS 109157	KJ718238	GQ180080	KJ718585	KJ718413	KJ718746		
